# The Sulphites in Typhoid or Enteric Fever

**Published:** 1868

**Authors:** R. S. Cross

**Affiliations:** Petersfield


### ARTICLE V.

*The Sulphites in Typhoid or Enteric Fever.* By R. S. Cross, Esq., M.R.C.S., L.S.A.

I beg to lay before the readers of *The Lancet* an account (abridged as much as practicable) of an outbreak of typhoid in the neighborhood of Petersfield, during the autumn of

1866. Its history commences with the return of a family from Mentone in the first week of July, one member of which, a little boy seven or eight years old, was only just convalescent from an an attack which had kept him there far into the hot season. From all I could gather, the disease was of a much more virulent kind than is usual in this country, the condition of the patient having been, in the later stages more especially, like that observed in low typhus; his father described the state of his tongue and mouth as if a coat of varnish had been applied and allowed to dry on it. Almost in despair of recovery, he was started homewards, and the change appeared to produce immediate improvement; for, although the weather was intensely hot all the way, and in London, where he remained a week after his arrival in England, he got home quite recovered. On the 27th we (Mr. Whicher and myself) were called to the lady's maid, a thin, weak-looking woman of about forty. Her symptoms were referred to the heat and fatigue of the journey after long watching and nursing at the bedside at Mentone. At this time, the end of her first week's indisposition, I confess to have had some suspicion of the true nature of her malady—a suspicion abundantly confirmed by the appearance of the same symptoms in the lady of the house a week later, in two of the maidservants at the end of another week, and then the gardener's wife (who had been called in to assist in the kitchen,) and also in the cook, butler, and others of the household—seven in all.

To go on with the history of the progress of the malady. At the end of six weeks, a woman from the parish, who had volunteered to wash the clothes in a cottage at the end of the garden, failed with it, and it was discovered afterwards that some blankets she had washed had somehow escaped disinfection. Three of her children took it in succession; then a man, his wife and five children, in a cottage some three hundred yards from where she lived, and so it spread; in all about thirty cases occurred, from the age of three to seventy-five. Most of the cases were very severe ones, the largest number appearing to have the malady in the most intense form. One boy, about nine years of age, in particular, had to learn to talk, etc., and seemed imbecile for some time. The aggregate duration of all the cases was ninety weeks.

The symptoms ushering in an attack were a worn expression of face, a sort of pinched look, great lassitude, pains in the head, back, and limbs, sometimes rigors, almost invariably diarrhœa, either with or without pains in the stomach and bowels, nausea or active vomiting, total loss of appetite, some thirst, sleeplessness, pulse quick, ranging between 90 and 120; tongue varying with the intensity of gastric symptoms; where active would be covered with a yellow coating, edges and tip dark red; where less intense, looked sodden, large and white; always tremulous; and this condition, together with the quick pulse and worn look, often existed many days, and almost weeks, before the patient complained very much, or gave up and took to bed. Nothing varied so much as the character and duration of the premonitory stage.

In one patient, the lady of the house, there was positively no warning. She became paler than usual, and so rapidly weaker and weaker, that on the third night of my attendance upon her, with a perfectly clean tongue, without diarrhœa or sickness, and in the entire absence of any dread of the disease, she was only saved from sinking by the unsparing use of brandy (two-thirds of a bottle this night), and such was the character of her illness throughout.

In another case, that of a stout plethoric girl of eighteen, the malady was ushered in suddenly by symptoms of most active biliary disorder, soon followed by the wildest delirium, requiring restraint for many days to keep her in bed even; both lung and intestinal complication following in the progress of the case, which was very long and doubtful. In many cases, the tongue was for days together dry and hard like toast, abdomen tympantic and tender, and the lungs the seat of frequently recurring congestion, engorgement and solidification, or whatever term may best describe a condition in which a very large portion of either one or both of these organs is often perfectly useless, if not worse; sore mouth, bleeding from the fauces, tongue, and gums, loss of the almost entire mucous covering, with deep ulcerated fissures, constituted a dreadful aggravation of suffering in numerous instances. The treatment up to August 19th had been such as is usually followed. In every case where indicated, a single dose of calomel, or an emetic, was administered at the outset, and always with advantage. At this period, not being much impressed with a belief in the power of any known mode of ordinary treatment to do much in combatting the disease, and not daring with six or eight people about me as good as dying to adopt the do-nothing plan, I determined to try the sulphites, the more especially after a careful perusal of the very able and exhaustive article from Dr. de Ricci in *Braithwaite's Retrospect*, vol. 49. I prescribed the sulphite of soda in scruple doses every four hours in water, adding for the most part quinine in grain doses as the purely gastric symptoms disappeared and were followed by the toast tongue, and other signs of greater encroachment on the vital powers. There was a liberal, very liberal, allowance of wine, brandy, and beef-tea given, and the quantity taken every twenty-four hours by most of the sufferers was truly astonishing. We had two good nurses from the Institution in London, besides other help. It was just pouring down the three things before named day and night to keep the flickering lamp of life alight; and, without a single exception, just in proportion as the hand was stayed from any cause, either accidentally in the nursing so many, or in obedience to an order from me, based on some fear or suspicion of over stimulation, so did the jeopardy of the patient immediately increase, and time and again it seemed hopeless that the fuller use of the brandy bottle could be in time to snatch the sufferer from the jaws of death. Some took a bottle of wine every twenty-four hours for three weeks at a stretch; and the way in which this was freely placed at my disposal by the fearless, intelligent master of the house, together with the champagne, ice, grapes, and everything that could be thought of, elicited my admiration, as much as his tact, discrimination, and appreciation of symptoms at the bedside by day and night, deserved my warmest thanks, as the greatest possible help in the management of such a visitation.

In the treatment of the diarrhœa, or, more properly speaking, the repeated loose action of the bowels, which was such an invariably prominent accompaniment, I did not adopt either the astringent or castor-oil plan in their entirety. Whether or not the increased action in question be due to the elimination by the glandular and mucous apparatus of the intestinal tract of a morbid poison from the blood, or the disease itself consist a primary affection of a specific kind, true it is, practically, that in every case there is, to begin with, a most serious invasion of the powers of life; and the cardinal point in treatment is not to allow the patient to either do or suffer anything whereby the jeopardy arising from this great exhaustion might be increased, at the same time avoiding a too active interference—an interference, I mean, amounting virtually to arrest of what, up to a certain point, may be a salutary process, and can be after all but one of many circumstances adverse to life. I prescribed now and then an astringent mixture, containing a small quantity of laudanum—in fact, the nurses were allowed to give a certain quantity of it to restrain a little when the actions were very frequent, particularly when occurring immediately after taking support of any kind. I am no stranger to the value of a dose of castor oil under some peculiar conditions of distended abdomen, etc.; but it is so nice a point I verily believe a patient's life often hinges upon the fine discrimination as to its immediate applicability to the case under observation. Turpentine stupes were always going either to the chest or abdomen; and they seemed as valuable in one place as the other, never failing to relieve alike the over-burdened and sometimes painful lung, and the distended, flatulent, and uneasy belly. The subject is practically inexhaustable; but I will bring this part of my remarks to a close by saying that there was, with the exception of the patient verging on eighty, who had the fever after nursing her daughter and family for weeks, and who finally succumbed to bed-sores, no death.

I also used the sulphite as a prophylactic; a bottle of mixture was established in the house, and given in scruple doses three times a day to five people. Of these five one was the constant attendant on the lady, two were in the kitchen, another was a keeper, who came in and out, and the butler. The attendant, though in the sick room night and day for weeks, never ailed anything. Of the two in the kitchen, one never ailed; the other, the cook, who was always running in and out the sick rooms, had all the symptoms of the malady,—the worn look, the sickness and purging, loss of appetite, quick pulse, characteristic tongue, etc., but never gave in; used to lie down and get up, but never was absent from her post a day. The keeper, the husband of the woman who took it by washing, was less affected, and also kept his post. The butler hung in the balance for a fortnight or tnree weeks, then took to his bed, and was among the worst.

Much as I believe it, I am hardly in a position to assert as much, but my experience powerfully endorses the opinion expressed by M. de Ricci in the *British and Foreign Medico-Chirurgical Review*, April, 1867, p. 523, wherein he predicts "that eventually the treatment of zymotic diseases by the administration of the sulphites will be as fully recognized as that of cinchona."

All I trust is, that after a careful perusal of this paper, I shall not be thought to have intruded these very imperfect and crude observations (recorded necessarily in the midst of much to divert) altogether uselessly upon the notice of my professional brethren, to many of whom an opportunity for making a similar trial may not only never have occurred, but be long wanting.

To proceed. On July 30th, 1867, I was requested to see Miss——, aged twenty, residing at a distance of eight miles in a country house, the inmates of which were, and always had been, particularly healthy. I was told it was the third day of her being in bed, and the history of the case pointed simply to more manifestation of fatigue of late, in the face, it is chanced, of more than usual call and inducement for exertion. A dose of oil had been given three days prior to my visit, and had done well. On entering her room, the position in bed, her manner and appearance, voice, and expression of face, all reminded one of the impression of fever. The pulse was weak and quick, over 100; tongue sticky and coated; breath offensive; no appetite, bowels uneasy, somewhat fuller and more tense than natural; no action since that from the oil. She was unable to sit up in bed without help, or to remain long so without faintness. Ordered to take ten grains of sulphite of soda every four hours, and pills at night containing a mild dose of mercury with chalk, rhubarb, ipecacuanha, and hyoscyamus. To have beef-tea; claret-and-water to be given freely as a drink.

July 31st (next day)—Condition essentially the same, but weaker; pulse quicker. Ordered claret without water. Pills had acted twice; feared to repeat them in the face of so much exhaustion. To continue the sulphite.

Aug. 1st—Much worse in every respect; pulse almost too quick to count, and the tendons of the muscles of the forearm were felt to twitch constantly under the finger, strongly enough to be seen also in other muscles; abdomen more distended, and slightly tender on pressure. Ordered turpentine stupe, a tablespoonful of brandy every hour in arrowroot, beef-tea, etc. Sulphite to be continued. Bowels rather relaxed.

2d—Much the same; in danger of sinking from hour to hour. Brandy increased to six drachms every two hours and the other two; two ounces of port wine to be given; beef-tea; sulphite continued. Bowels the same; slight cough. Remained the night.

With very little alteration, this condition prevailed up to the 9th, when, the bowels acting rather more frequently than I liked—the ingesta quickly passing through, almost unchanged,—I ordered a pill at night, containing half a grain of opium, half a drop of creasote, and one grain of camphor.

The chest symptoms had fairly set in, there being complete dulness on percussion and entire absence of respiratory murmur over the lower half of left lung, with slight pain, and that coarseness of breath-sound on the right side which I have noticed to be the sure forerunner of that alteration in the lung tissue which occurs in connexion with the disease in question, as well as not unfrequently constituting a separate and distinct malady. Both sides of her chest were thus affected, and the turpentine stupes were applied again and again, always with marked benefit. The abdominal symptoms had appeared to decline from the first application of turpentine, and especially from the date of appearance of chest symptoms; nor had the tongue been dry and baked for more than twenty-four hours before time sufficed for getting the system under the influence of the maximum quantity of stimulus. The creasote-and-opium pills were given occasionally, eight in all.

At this period—the end of the fortnight—an ounce of the sulphite in all had been taken. And with regard to the time of its continued exhibition, I am disposed to think it should be dependant on the period of the malady at which it is begun, and the power it manifests to control the progrest of symptoms. M. de Ricci says that "the want of success which has sometimes been observed in the treatment of zymotic diseases by the alkaline and earthy sulphites is attributable to the fact that these remedies have not been administered early enough. If the treatment is too long delayed, the blood becomes so loaded with poison and deteriorated in quality as to be no longer able to perform its normal function ; and then the sulphites are of no more service than any other remedies, because they cannot restore to life the dead blood-corpuscles. The sulphites should, therefore, be administered early, while still a large portion of the blood is in a healthy state, and capable not only of carrying on life, but of throwing off what has been rendered inert by the presence of the sulphuric acid." M. de Ricci attributes another source of failure to "the administration of hyposulphite of soda instead of the sulphites, and especially the sulphite of magnesia. The hyposulphite of soda is less efficacious than the sulphites, because, in the former, the greater part of the acid becomes oxidised in its passage through the animal economy, and appears in the urine as a sulphate ; because, being a salt of hyposulphurous acid, it is a less active antagonistic ; and because it often causes a troublesome diarrhœa : while the sulphites of soda and magnesia never produce such effects." And so on in the article in question.

To return to my patient. I discontinued, then, the sulphite at the end of the fortnight, and ordered a mixture of chlorate of potash, ammonia, cinchona, and chloric ether on the 14th, which was continued to the 20th. Somewhat later than this, the pulse having improved, and finding her at my visit somewhat flushed, feverish, and excited, I ventured to diminish one-half of her stimulants; but I was only too glad to put it on again the next day, and lost three days most certainly by the experiment. And it appears to me most important to be very cautious about reducing the allowance of wine, etc., allowing the patient to sit up in bed or make any exertion, or to take solids too soon. Castor oil was given twice : the first time at the end of a month from the time of her attack; and she was able to be down stairs and take carriage exercise at the end of six weeks, from which point her recovery was rapid and complete.

The other case I was called to on September 15th. I will be brief with it. A young man of twenty, stout, florid complexion, was a draper's assistant at a neighboring seaside town. Had been ill three days. He was prescribed for, the medicine purging him freely. Had been ill a week altogether at the time of my being called to him after his removal home. He had all the symptoms of a severe attack of the fever in question. The lung complication was thus early extreme on both sides. He became breathless on the slightest exertion, and almost fainted on my attempting to set him up to examine his chest posteriorly. Ordered turpentine stupes, sulphite, beef-tea, wine, etc. The case went on to the 27th, when he got rather suddenly much worse, I believe from neglect in the administration of support and stimulus. He was restless, with wandering delirium, dilated pupils, confusion about the head, etc. The mouth and fauces were covered with a thick coat of aphthous exudation. These parts were sponged out well with a solution of the biborate of soda, the hair was removed, and a mustard plaster applied to the nape of the neck; and he was ordered to take in the course of the next twenty-four hours a bottle of wine, and a tablespoonful of brandy occasionally. The next day he was much better in every respect, and continued to mend from that time, making an excellent recovery, and passing out of our hands well on the 16th of October: just one month ill. He took in all two ounces of the sulphite.

I could give a later case, in which the very early use of the remedy seemed to cut short the malady, and reduced the term of indisposition to but a fortnight altogether; but I forbear, having already, I fear, tresspassed at too great length.

I should have mentioned that in all cases I had Burnett's solution kept in the utensils used by the invalids, these being used immediately after use. Chloride of lime was kept going under the beds, and about the rooms and passages. I insisted on as much air as could be got, without placing the patient in a thorough draught. One of the quickest and best recoveries of the very bad cases occurred in the gardener's wife, whose bed was so situated (in a small room without a fireplace) that the wind blew in at the window upon her day and night; another, the boy before alluded to, the window of whose room was clean gone, a sack hung up doing duty for one, but which I often found down, admitting the wind, and sometimes the rain, on to the little fellow's bed, which was shared by a brother ill of the same disease.

I would observe, in conclusion, that to many the foregoing remarks may read more like an advocacy of the ultra-stimulating plan of treatment than that by the sulphites. To such I would say is it not necessary in all maladies to maintain the powers of life in such a way as shall give time and opportunity for remedies to take effect? and is not the degree of energy with which this support is applied in direct proportion to the patient's need? The quantity of stimulus must not be measured by spoonfuls, but by the observation of how much is required to keep the patient alive and in a likely condition to be benefitted by all other adjuvants, chemical and otherwise.—*London Lancet.*

Petersfield, Dec. 1867.

